# A validated stability-indicating TLC-densitometric method for the determination of stanozolol in pharmaceutical formulations

**DOI:** 10.1186/1752-153X-7-142

**Published:** 2013-08-27

**Authors:** Syed Ghulam Musharraf, Qamar ul Arfeen, Wardah Mazhar, Nayab Kanwal

**Affiliations:** 1 H.E.J. Research Institute of Chemistry, International Center for Chemical and Biological Sciences, University of Karachi, Karachi 75270, Pakistan; 2 Department of Chemistry, University of Karachi, Karachi 75270, Pakistan

**Keywords:** Stanozolol, TLC-densitometry, Stress degradation

## Abstract

**Background:**

Stanozolol is a synthetic derivative of dihydrotestosterone (DHT), and one of the frequently detected anabolic steroids in doping analysis. The current work describes the development and validation of the stability-indicating TLC-densitometric method for sensitive and specific estimation of stanozolol even its degradation product being there. Precoated silica gel TLC aluminium plates were utilized as the stationary phase and the eluent comprised of petroleum ether: acetone (6:4, v/v). Densitometric analysis of stanozolol was achieved at *λ*_max_ 750 nm in the absorbance mode after staining with phosphomolybdic acid (PMA). Stress degradation of stanozolol was carried out under various reaction conditions including acid, base and neutral hydrolysis, wet and dry heating treatment, oxidation, and photo-degradation. Resulted stress samples and pharmaceutical products were analyzed with the developed TLC method.

**Results:**

This system showed a compact spot for stanozolol at *R*_f_ value of 0.46 ± 0.02. The data of linear regression analysis indicated a good linear relationship over the range of 200–1200 ng/spot concentrations. The method was validated for robustness, precision and recovery. The LOD and LOQ were 1.6 and 5.1 ng/spot, respectively. Under various stressed conditions, stanozolol showed degradation only under acidic hydrolysis. Peak of a degraded product was well resolved from the stanazolol with reasonably different *R*_f_ value and identified as 17, 17-dimethyl-l8-nor-5α-androst-13(14)-eno [3,2c] pyrazole through 1D- and 2D-NMR spectroscopic techniques and ESI-QqTOF-MS/MS analysis.

**Conclusion:**

Result reflected that the stanozolol is majorly affected by the acidic condition. Statistical analysis indicated the application of the developed stability-indicating TLC-densitometric method for routine analysis of stanozolol in the presence of its degradation product.

## 

Stanozolol (5α-androstane-17α-methyl-17β-ol [3,2-c] pyrazole), a heterocyclic anabolic androgenic steroid was first synthesized by Clinton in 1959 [1]. It differs in structure with endogenous steroid hormones and most commonly used exogenous anabolic steroids [2]. Despite the restriction by the International Olympic Committee (IOC) since 1974, stanozolol is one of the most frequently misused synthetic anabolic steroids in sport. Several authors reported it’s used in the treatment of osteoporosis and deficiency in protein synthesis [3-5]. It is also used in veterinary medicine to boost appetite, to weight gain, and for the treatment of certain types of anemia [6,7].

In order to develop stability indicating methods, stress degradation study is a powerful tool used routinely in pharmaceutical development that lead to a quality stability data and to recognize the degradation pathways of the active drug ingredient and its products. These degradation studies are employed for monitoring the drug stability which provides evidence that in what manner drug quality varies with passage of time by the consequence of different environmental factor including temperature, light, humidity, etc. and effect of stomach and intestine pH. Various strategies and principles are provided by International Conference on Harmonization (ICH) and the FDA for conducting forced degradation studies [8-10].

Different analytical techniques such as gas chromatography–mass spectrometry (GC-MS), high performance liquid chromatography-tandem mass spectrometry (HPLC-MS/MS), high throughput ultra performance liquid chromatography-tandem mass spectrometry (UHPLC-ESI -MS/MS) have been described for the analysis of stanozolol in variety of biological samples [11-14]. However, there is no report for the development of stability-indicating TLC-densitometric method for the quantification of stanozolol in the presence of its degraded products so far. Currently, TLC-densitometric method getting considerable attention on account of its various benefits including simultaneous running number of samples, utilizing little amount of eluent in contrast to HPLC, reduced cost and analysis time, mobile phase of extreme pH and direct application of various type of samples (dirty, turbid or suspensions) can also be employed [15].

In continuation of our studies on the determination of organic compounds of biological interest and pharmaceutical importance [16-19], and taking ICH guidelines Q1A in consideration, present study describes a simple stability-indicating TLC densitometric method for the quantification of stanozolol in presence of its degraded products formed under the applied stress conditions.

## Experimental

### Standards and chemicals

Standard stanozolol was purchased from Sigma Aldrich (USA). Stanozolol® tablets (containing 5 mg stanozolol/tablet (S1) and 10 mg stanozolol/tablet (S2) manufactured by LA Pharma S.r.I) were procured from the local pharmacies of Karachi, Pakistan. Aluminum sheets precoated with silica gel (60F-254, 20 cm × 20 cm) were purchased from the Merck (Germany). Hydrochloric acid (HCl) and hydrogen peroxide (H_2_O_2_, 35% v/v) were purchased from the Fisher Scientific (UK) while sodium hydroxide was procured from the BioM Laboratories (Cerritos, USA). Deionized water was acquired from Millipore Milli Q Plus System (Bedford, USA). All additional analytical grade reagents and chemicals were procured from Merck (Germany).

### Chromatographic state and instrumentation

A CAMAG automatic TLC sampler (Linomat 5) was used for the spotting of all samples and standards, which were spotted in the form of bands of width 6 mm with a CAMAG 100 μL syringe on pre-coated silica gel TLC aluminum sheets. CAMAG Reprostar 3 was used for the video densitometry of the chromatoplate, and the integrated software of WinCATS was used for the analysis. Sample was applied at the constant rate of 0.1 μL/s and 9.1 mm space between the two bands was maintained. The eluent composed of petroleum ether and acetone (6:4, v/v). 20 cm × 10 cm CAMAG twin trough glass chamber was used for linear ascending development of TLC plate under unsaturated condition and 10 mL of organic solvent was consumed per run. The TLC plate was developed up to a distance of 80 mm. Chromatographic process was carried out at room temperature (25 ± 2°C) with relative humidity of 42 ± 5%. The developed TLC plate was dried with the help of air dryer for 5 min and subjected to the staining process using CAMAG Chromatogram Immersion Device. Phosphomolybdic acid (PMA) was used to visualize the analyte. The analyte complexed with PMA was then heated with CAMAG TLC Plate Heater 3 at 120–125°C for 3–4 minutes. CAMAG TLC Scanner 3 was used for scanning at λ_max_ 750 nm and operates in reflection absorbance mode by winCATS software. Each sample and standard level was spotted in triplicate, and during scanning baseline correction was employed. Evaluation was accomplished via peak areas using linear regression.

### Calibration curve of stanozolol

Stock solution of stanozolol (1 mg mL^-1^) was prepared in methanol. The stock solution was further diluted to obtain a series of working standards over the range of 200–1200 ng spot^-1^ for calibration curve. Prepared solutions were stored at 4°C until use. Each working standard was spotted in triplicate, total 18 tracks were spotted on each TLC plate. The spot volume of each standard level was 5 μL. The spotted plate was developed as mentioned in previous section. This practice was repeated six times to get an average standard calibration curve. Linear least- square regression was performed on the data of peak areas plotted versus the corresponding concentrations.

### Validation of method

The method validation was done as described by the ICH guidelines. The method sensitivity was estimated with respect to limit of detection (LOD), limit of quantification (LOQ) and correlation coefficient. Working solutions containing 200–1200 ng of stanozolol were spotted on TLC aluminum sheet. In order to evaluate LOD and LOQ, calibration curve was used and were evaluated by using following equation: LOD = 3.3 δ/S, LOQ = 10 δ /S where, S = the slope of the calibration curve, δ = the residual standard deviation of regression line or the Y-intercept of regression line. The LOD and LOQ were estimated as 3 and 10 times of the noise level, correspondingly. Moreover, both were tentatively determined by diluting the familiar concentration of stanozolol standard till the mean instrumental responses were nearly three and ten times of the standard deviation of the responses for six manifold analyses.

The Intra- and inter-day variation for the estimation of stanozolol was evaluated for method precision. It was achieved by using three different concentration levels of 300, 500 and 700 ng spot^-1^. Repeated analyses were carried out in a same day for intra-day analysis while the same practice was repeated next day for inter-day analysis. Intra- and inter-day analyses were performed to check the repeatability and reproducibility of the method, respectively and results were statistically evaluated in terms of % R.S.D. In order to check the robustness, following parameters were intentionally changed within the range of ± 5% at 300, 500 and 700 ng/ spot concentration level; mobile phase composition, different types of TLC plates e.g., silica-gel glass plates and aluminum sheets of other supplier (Macherey-Nagels, Germany), time from spotting to chromatography, time from chromatography to staining, time for staining and time from staining to scanning. The accuracy of the method was assessed by performing recovery study of pre-analyzed samples, spiked with extra 50, 100 and 150% standard stanozolol. The analysis of spiked samples was repeated six times.

### Preparation and analysis of pharmaceutical products

To estimate the stanazolol content in pharmaceutical products, the tablets (label claim: 5 mg/tablet, 10 mg/tablet) were crushed and amount equivalent to one tablet was extracted in 2 mL methanol. To confirm the complete extraction of the stanazolol, it was shaken at 40°C for 20 minutes in a thermomixer (Eppendorf® Thermomixer Comfort, UK) at 300 rpm. The resulting solution was filtered with glass syringe with disposable micropore filter tips (13 mm × 0.45 μm Teflon filter) and the filtrate was used for the drug content analysis. From the filtrate, 5 μL was spotted onto the plate and then development, staining and scanning were done as previously described. This was repeated in triplicate.

### Preparation of forced degradation products

Stress degradation studies of stanozolol were performed using parallel synthesizer (Smart Start Synthesizer, Chem Speed Ltd., Switzerland). Stock solution (1 mg mL^-1^) was used for forced degradation. After the completion of reaction, all the resulting solutions were preserved at −20°C prior to analysis. Average peak areas of active components were analyzed after triplicate analysis. For acidic hydrolysis, 0.1N, 1N and 5N HCl were used, for alkaline hydrolysis, 0.1N, 1N and 5N NaOH were used while for neutral hydrolysis Milli Q water was used. 3 mL of each concentration of acidic and alkaline solutions and Milli Q water were added into 3 mL (1 mg/mL) stock solutions of each. To study wet heating degradation, 3 mL (1 mg/mL) of stock solution was used. Oxidation was carried out by adding 3 mL of H_2_O_2_ (35% v/v and 5% v/v) in 3 mL stock solution of each set. All the resultant solutions were refluxed for two hours at 80°C in parallel synthesizer in the dark, in order to prohibit the possible degradative effects of light. 2 μL (1000 ng/spot) of 0.1N, 1N and 5N HCl treated solution, 0.1N, 1N and 5N NaOH treated solutions, and neutral hydrolysis solution while 1 μL (1000 ng/spot) of wet-heating degradation mixture and 2 μL (1000 ng/spot) from oxidation mixture solution were applied on TLC plate in triplicate and densitogram were developed.

Dry heating degradation was conducted by taking standard stanozolol and heated in oven at 90°C for 6 hrs. Photochemical degradation was conducted by taking standard stanozolol and placed in direct sunlight for three days from 8 to 18 hrs at 30 ± 2°C. 1 mg of each treated standard was dissolved in 2 mL of methanol and 2 μL (1000 ng/spot) of each resultant solution was spotted on TLC plate in triplicate for chromatographic analysis. For oxidation reaction at room temperature, 3 mL stock solution of stanozolol was added with 3 mL of H_2_O_2_ (35% v/v) and the resultant solution was kept for 24 hours at room temperature. 2 μL (1000 ng/spot) of treated solution was applied on TLC plate in triplicate for chromatographic analysis.

### Isolation and characterization of acid degradation product

In order to isolate the acid degradation product in bulk amount, 500 mg of stanozolol in methanol (1 mg/mL) was refluxed in 1N HCl for 3 hours and monitored by TLC analysis (petroleum ether: acetone; 6:4, *v/v*). At the end of reaction, the mixture was neutralized with sodium bicarbonate solution and extracted with dichloromethane. The reaction mixture was loaded on to a silica gel column, which on elution with hexane: ethyl acetate; 6.5:3.5, v/v afforded purified degradation product (110 mg; 22% yield) which was identified with the aid of 1D- and 2D-NMR spectroscopic techniques and ESI-QqTOF-MS/MS analysis.

## Results and discussion

### Method optimization

**Figure 1:**
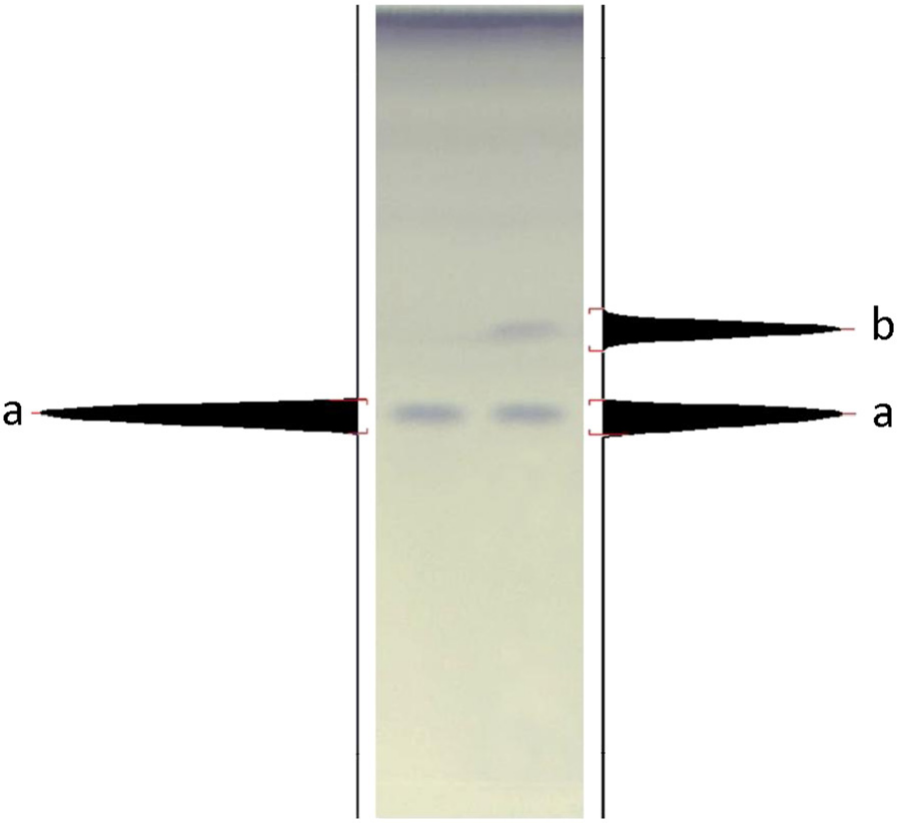
**HPTLC chromatogram of standard stanozolol (800 ng/spot) and acid treated (1N HCl, reflux for 2 hr at 80°C; 1000 ng/spot) after staining with phosphomolybdic acid (PMA), stanozolol peak has *R***_**f **_**= 0.46 ± 0.02 (a) and degradant has *R***_**f **_**= 0.55 (b): mobile phase; petroleum ether: acetone (6:4, v/v).**

For the development of chromatographic method, the mobile phase composition was optimized with a view to develop stability-indicating assay method. TLC sheet was simultaneously spotted with standard stanozolol, pharmaceutical samples and degraded products and was developed in different solvent systems. Various mobile phases were tried to resolve stanozolol from its degraded products. *R*_f_ values and peak widths of stanozolol in different mobile phases are summarized in Table 1. Suitable separation with best resolution was achieved with petroleum ether and acetone (6:4, v/v). This system showed sharp and symmetrical peaks of stanozolol and its acid degradation product under unsaturated condition with *R*_f_ value at 0.46 ± 0.02 and 0.55 ± 0.01 respectively, (Figure 1) with 1.5 resolution. Various derivatizing agents like ceric sulfate, vanillin, phosphomolybdic acid (PMA) and ceric ammonium sulfate were tried. However, derivatization with phosphomolybdic acid (PMA) gave significantly better result, the spots are visible, sharper and more colourful as compared to the plate sprayed with other derivatizing agents described above.

**Table 1:** ***R***_**f **_**values and peak widths of stanozolol in different mobile phases**

**S. No.**	**Compositions (v/v)**	**Proportions**	***R***_**f**_	**Peak widths (cm)**
1	Petroleum ether-acetone	8:2	0.24	0.07
2	petroleum ether-acetone	7.5:2.5	0.29	0.09
3	Petroleum ether-acetone	6:4	0.46	0.06
4	Hexane-ethyl acetate	8:2	0.05	0.06
5	Chloroform-methanol	9:1	0.17	0.09
6	Dichloromethane-methanol	9.2:0.8	0.13	0.09

The linear regression data for the calibration curves (n = 6) showed a good linearity r = 0.9990 ± 0.0004, (Slope = 5.841 ± 0.755, Intercept = 1420.3 ± 36.4) over the concentration range of 200–1200 ng/spot with respect to peak area. In residual linearity test of stanozolol, the random pattern of residuals against respective standard levels showed a linear model of standard calibration curves Additional file 1: Figure S1).

### Method validation

The repeatability and reproducibility were showed in terms of percentage relative standard deviation (R.S.D. %) between two analyses and was found to be < 2% (Table 2). Similarly, intra- and inter-day variation of stanozolol at three different concentration levels 300, 500 and 700 ng/spot was found to be < 2% in all the cases (Table 2).

**Table 2:** Precision and accuracy for quality control standard of stanazolol

				**Analyst 1**			**Analyst 2**		
**Intra-day**				**Inter-day**					
**Conc.**	Found^*^	**R.S.D.**	**Accuracy**	Found^*^	**R.S.D.**	**Accuracy**	Found^*^	**R.S.D.**	**Accuracy**
**(ng)**	**(ng)**	**%**	**(%)**	**(ng)**	**%**	**(%)**	**(ng)**	**%**	**(%)**
300	303.56 ± 5.00	1.65	98.81	306.63± 5.52	1.80	97.79	300.62± 1.452	0.48	99.79
500	503.33 ± 1.06	0.21	99.33	504.49± 3.38	0.67	99.10	502.63± 0.78	0.15	99.47
700	705.80 ± 1.50	0.21	99.17	703.85± 1.14	0.16	99.45	703.04± 0.71	0.10	99.56

^*^Values are mean of n = 3.

The standard deviation of % yield of three standard levels (300, 500 and 700 ng) was estimated for each parameter for robustness analysis. Mean % R.S.D. was 2.4 for varying in mobile phase composition, 1.39 for the use of different TLC plates, 2.27 for varying time from spotting to chromatography, 1.26 for varying time from chromatography to staining, and 1.77 for varying time for staining and 2.1 for varying time from staining to scanning (Table 3). RSD % is reasonably low (< 2.5%) for all parameters which indicates the robustness of the developed method. The LOD for standard stanozolol with signal/noise ratio of 3:1 was found to be 1.6 ng per spot and LOQ with signal/noise ratio 10:1 was found to be 5.1 ng/spot. This showed the satisfactory sensitivity of the developed method. The spectra were compared at peak start, peak apex and peak end positions of the spot, to check the peak purity of stanozolol. Excellent correlation, *r*^2^ (start to middle) = 0.9996 and *r*^*2*^ (middle to end) = 0.9998 was observed by comparing the spectra of standard and corresponding peak in pharmaceutical samples. Spiking with 50, 100 and 150% of additional standard stanozolol afforded recovery of 92.2–95.2% as showed in Table 4. This practice was repeated six times. Summary of all validation parameters is highlighted in Table 5.

**Table 3:** Robustness testing (n = 6)

**Standard level**	**Mobile phase composition**		**Nature of TLC**		**Time from spotting to chromatography**		**Time from chromatography to staining**		**Time for staining**		**Time from staining to scanning**	
**Amount (ng/spot**^**-1**^**)**	**Amount detected (ng ± SD)**	**R.S.D. (%)**	**Amount detected (ng ± S.D.)**	**R.S.D. (%)**	**Amount detected (ng ± SD)**	**R.S.D. (%)**	**Amount detected (ng ± SD)**	**R.S.D. (%)**	**Amount detected (ng ± SD)**	**R.S.D. (%)**	**Amount detected (ng ± SD)**	**R.S.D. (%)**
300	303.7 ± 11.54	3.80	298.3 ± 1.54	0.51	311.29 ± 8.03	2.58	284.18 ± 6.81	2.39	300.63 ± 9.34	3.11	287.3 ± 10.06	3.5
500	505.01 ±12.44	2.46	500.7 ±13.58	2.71	498.51 ± 18.85	3.78	513.85 ± 6.91	1.34	516.18 ± 2.58	0.50	508.53 ± 12.96	2.54
700	705.23 ± 6.64	0.94	698.46 ± 6.64	0.95	717.03 ± 3.43	0.47	699.61 ± 0.51	0.07	714.95 ± 12.30	1.72	704.29 ± 3.33	0.47

**Table 4:** Recovery studies (n = 6)

**Sample**	**Standard spiked %**	**Theoritical ng spot**^**-1**^	**Mean recovery %**	**RSD %**
	50	351.5	92.2	5.00
S1	100	468.7	95.2	5.99
	150	585.9	94.6	1.76

**Table 5:** Summary of validation parameters

**Parameter**	**Data of standard stanazolol**
	**(at *λ***_**max **_**750 nm)**
Linearity range	200-1200 ng/spot
Correlation coefficient	0.9990 ± 0.0004
Limit of detection (LOD)	1.6 ng/spot
Limit of quantification (LOQ)	5.1 ng/spot
Y = mx + c	Y = 5.841x + 1420.3
Slope ± SD	5.841 ± 0.755
Intercept ± SD	1420.3 ± 36.4
Intra-day analysis (n=3),	0.690
% R.S.D.	
Inter-day analysis (n=3),	0.877
% R.S.D.	
Robustness	Robust
Specificity	Specific

### Analysis of commercial dosage form

In chromatogram of tablet’s extracted samples, a single spot (*R*_f_ value of 0.46 ± 0.02) was observed corresponding to stanazolol. No interference from the excipients and binders commonly present in the marketed tablet formulation was observed. Two different dose strengths, 5 and 10 mg were analysed through developed method and it showed drug content of 5.198 ± 0.16 mg and 10.126 ± 0.25 mg, respectively. The mean percent recovery of the proposed method is 103.96 for S1 and 101.26 for S2. The low R.S.D. % value indicated the suitability of this method for routine analysis of stanozolol in pharmaceutical products.

### Stability-indicating property

**Scheme 1:**
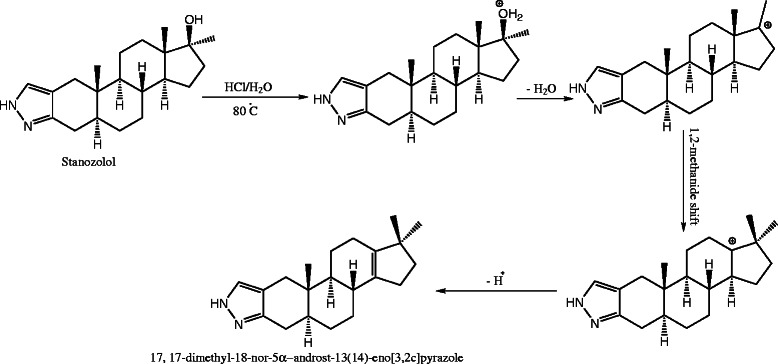
Proposed acid degradation pathway of stanozolol.

Stanazolol was subjected to various stress degradation conditions. The chromatogram of stanozolol treated with different strength of acid showed well-separated peak of stanozolol as well as one additional peak of degradant (*R*_f_ = 0.55) (Figure 1). Stanazolol showed 11.7%, 23.7% and 54.9% degradation under 0.1N HCl, 1N HCl and 5N HCl conditions, respectively. Very low degradation was also observed after exposing the stanozolol to stressed conditions of base, and neutral hydrolysis, wet and dry heating, sunlight and oxidation. However, peaks of degraded products were not observed. Results of the stress degradation studies are summarized in Table 6. Acid degradation product was purified and characterized through various spectroscopic methods as 17, 17-dimethyl-l8-nor-5α-androst-13(14)-eno [3,2c] pyrazole which was earlier reported as a synthetic derivative of stanozolol [20]. The HRESI-MS (positive mode) showed peak [M+H]^+^ peak at *m/z* 311.2452 corresponding to C_21_H_31_N_2_ (calc. 311.2481), 18 amu less than stanozolol, indicating a dehydrated product. The ESI-MS/MS analysis of *m/z* 311 [M+H]^+^ showed base peak at *m/z* 81, corresponding to pyrazole ring (Additional file 1: Scheme S1). The product ion at *m/z* 95 is suggested to derive from the same protonated species as *m/z* 81 by a rearrangement of the bonds. Fragment due to retro Diel-Alder cleavage was observed at *m/z* 189 from fragments *m/z* 311. The CID-MS/MS spectra and proposed mechanistic fragmentation pathway are shown in Additional file 1: Figure S2 and Additional file 1: Scheme S1, respectively and the characteristic MS/MS fragments are summarized in Additional file 1: Table S2. Proposed mechanism for the acid degradation of stanozolol is shown in Scheme 1. ^13^C-NMR and ^1^H-NMR data of a degraded product is provided in Additional file 1: Figure S3 and Additional file 1: Table S1.

**Table 6:** Summary of stress degradation studies of stanozolol

**Degradation conditions**	**% Recovery**	**% Degradation**	***R***_**f **_**of degraded products**
Acidic hydrolysis ^a^			
0.1N HCl	88.28	11.72	0.49, 0.55
1N HCl	76.25	23.75	0.49, 0.55
5N HCl	45.07	54.93	0.49, 0.55
Basic hydrolysis ^a^			
0.1N NaOH	98.40	1.60	Not detected
1N NaOH	95.19	4.81	Not detected
5N NaOH	90.71	9.29	Not detected
Neutral hydrolysis ^a^	98.65	1.35	Not detected
Wet heating ^a^	99.59	0.41	Not detected
Dry heating	98.32	1.68	Not detected
Oxidation ^a^			
5%v/v H_2_O_2_	97.90	2.10	Not detected
35%v/v H_2_O_2_	99.59	0.41	Not detected
Oxidation at room temp	98.30	1.70	Not detected
Photostability-daylight	98.62	1.38	Not detected

^a^Reflux in parallel synthesizer for two hours at 80°C.

## Conclusion

A new stability-indicating TLC method combined with densitometry has been developed and validated for the identification and quantification of stanozolol in its pharmaceutical dosage form. Satisfactory precision and accuracy, minimum cost, and quicker analysis are the core features of this method. The ICH guideline was followed for method validation. Reproducibilty and specificity of method was determined by statistical analysis. Moreover, this study demonstrates the degradation susceptibility of stanozolol to different stress conditions and thus helps in determining the changes in chemical and physical properties of the stanozolol with the passage of time. Current study may be expanded to degradation kinetics of stanozolol and for its assessment in urine, plasma, serum and other biological fluids.

## Competing interests

Authors declare that they have no competing interests.

## Authors’ contributions

SGM: Participated in the experimental designing and method optimization. QA: Performed the experiments and wrote the manuscript. WM and NK: Participated in experimental work and also involved in the useful discussion. All authors read and approved the final manuscript.

## Supplementary Material

Additional file 1: Figure S1 Standard calibration curve (A) and its residual linearity test (B). **Figure S2** CID-MS/MS spectra of acid-degradation product of stanozolol at the collision energy of 45 ev. **Figure S3** 13C-NMR and 1H-NMR (400 MHz, CDCl3) spectra of acid degradation product of stanozolol **Table S1** 13C-NMR and 1H-NMR (400 MHz, CDCl3) chemical shifts of acid degradation product of stanozolol (δ in ppm). **Table S2** Elemental composition of daughter ions of acid degradation product of stanozolol (m/z 311). **Scheme S1** Proposed CID-MS/MS fragmentation pathway of acid-degradation product of stanozolol. Click here for file
