# Supplementary material for: A validated stability-indicating TLC-densitometric method for the determination of stanozolol in pharmaceutical formulations
Source: Chem Cent J. 2013 Aug 27;7:142. doi: 10.1186/1752-153X-7-142 (PMC3766227; doi:10.1186/1752-153X-7-142)
Supplement: Additional file 1: Figure S1 — Standard calibration curve (A) and its residual linearity test (B). Figure S2 CID-MS/MS spectra of acid-degradation product of stanozolol at the collision energy of 45 ev. Figure S3 13C-NMR and 1H-NMR (400 MHz, CDCl3) spectra of acid degradation product of stanozolol Table S1 13C-NMR and 1H-NMR (400 MHz, CDCl3) chemical shifts of acid degradation product of stanozolol (δ in ppm). Table S2 Elemental composition of daughter ions of acid degradation product of stanozolol (m/z 311). Scheme S1 Proposed CID-MS/MS fragmentation pathway of acid-degradation product of stanozolol. [file 1752-153X-7-142-S1.docx]

**Additional file1**

**Figure S1** Standard calibration curve (A) and its residual linearity test (B).

**Figure S2** CID-MS/MS spectra of acid-degradation product of stanozolol at the collision energy of 45 ev**.**

**Figure S3** ^13^C-NMR and ^1^H-NMR (400 MHz, CDCl_3_) spectra of acid degradation product of stanozolol

**Table S1** ^13^C-NMR and ^1^H-NMR (400 MHz, CDCl_3_) chemical shifts of acid degradation product of stanozolol (*δ* in ppm).

**Table S2** Elemental composition of daughter ions of acid degradation product of stanozolol (*m/z* 311).

**Scheme S1**. Proposed CID-MS/MS fragmentation pathway of acid-degradation product of stanozolol.

**Figure S1** Standard calibration curve (A) and its residual linearity test (B).


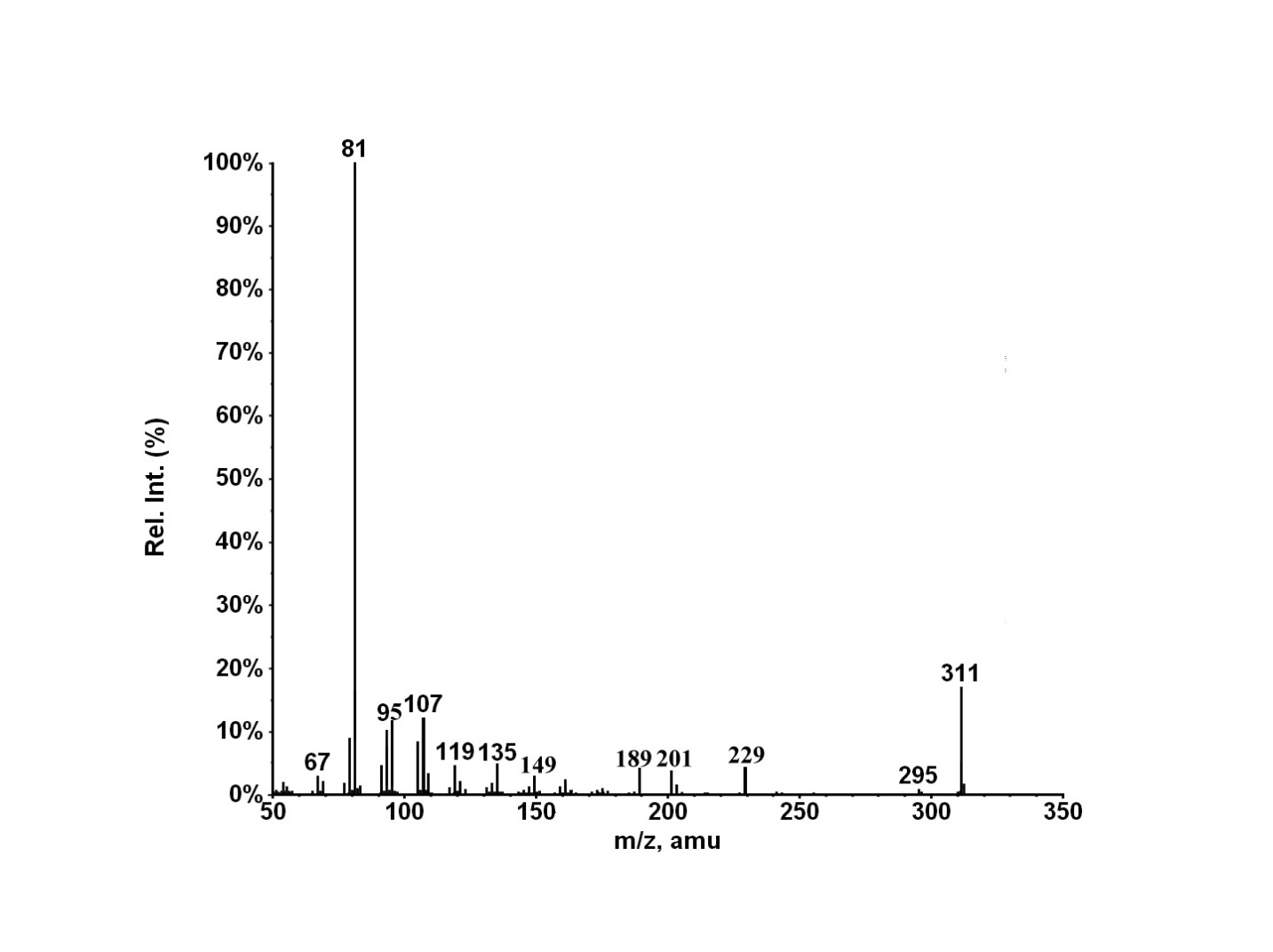

**Figure S2** CID-MS/MS spectra of acid-degradation product of stanozolol at the collision energy of 45 ev**.**

**
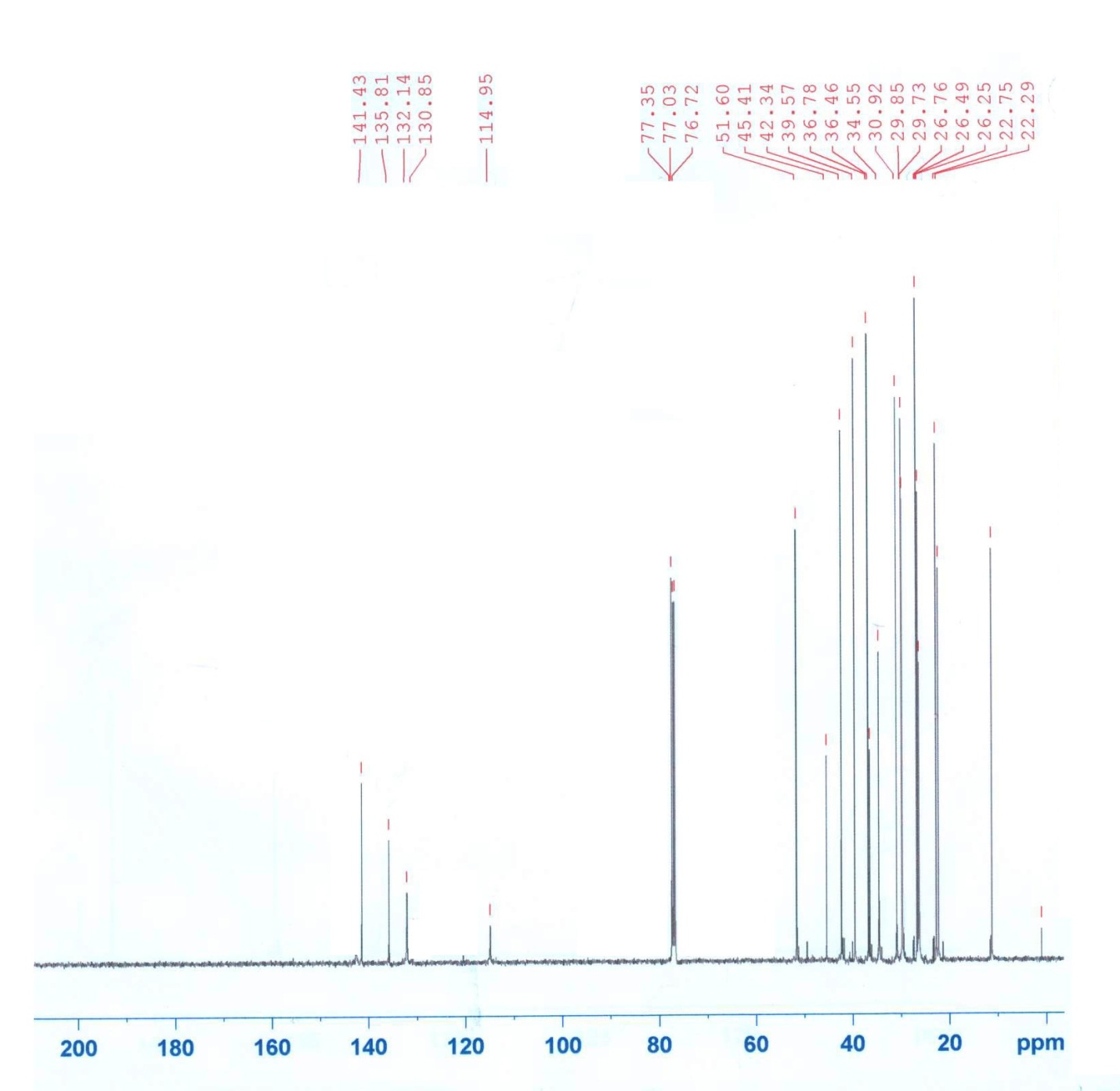
**

**
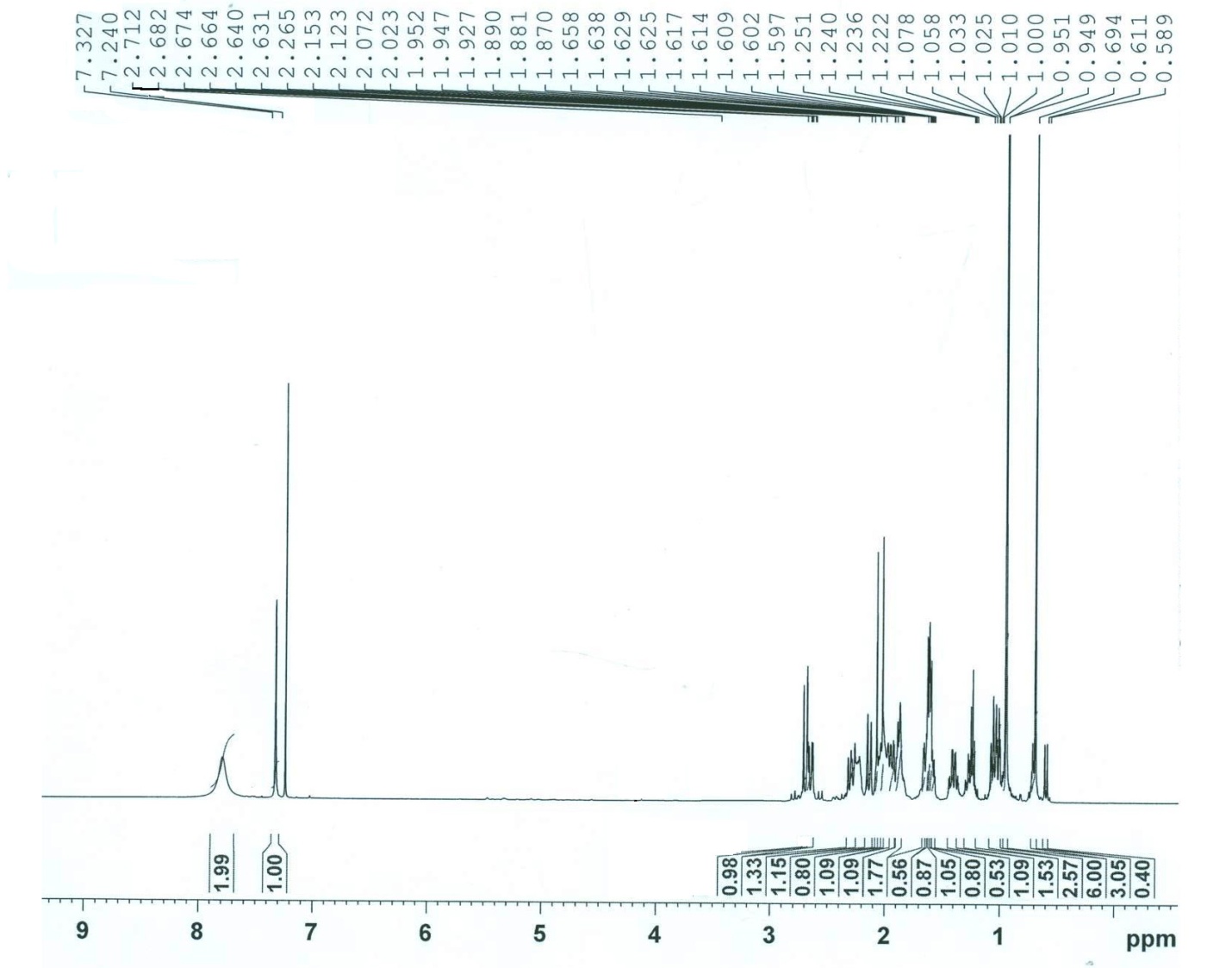
**

**Figure S3** ^13^C-NMR and ^1^H-NMR (400 MHz, CDCl_3_) spectra of acid degradation product of stanozolol

**Table S1.** ^13^C-NMR and ^1^H-NMR (400 MHz, CDCl_3_) chemical shifts of acid degradation product of stanozolol (*δ* in ppm).

| C.NO. | Number of protons attached ^a,b^ | ^13^C-NMR | ^1^H-NMR |
| --- | --- | --- | --- |
| 1 | CH_2_ | 34.55 | 2.70 (1H, d *J* = 15 Hz), 2.15 (1H, d *J* = 15 Hz) |
| 2 | C | 113.2 | - |
| 3 | C | 131.2 | - |
| 4 | CH_2_ | 25.75 | 2.67 (1H, dd *J* = 15.6, 9.4 Hz), 2.31 (1H, dd *J* = 15.7, 9.4 Hz) |
| 5 | CH | 42.34 | 1.6 (1H, m) |
| 6 | CH_2_ | 29.85 | 1.4, 1.63 (2H, m) |
| 7 | CH_2_ | 30.9 | 1.95, 1.02 (2H, m) |
| 8 | CH | 36.79 | 2.02 (1H, m) |
| 9 | CH | 51.6 | 1.07 (1H, m) |
| 10 | C | 36.4 | - |
| 11 | CH_2_ | 22.75 | 1.27, 1.24 (2H, m) |
| 12 | CH2 | 22.29 | 2.02, 1.86 (2H, m) |
| 13 | C | 141.43 | - |
| 14 | C | 135.81 | - |
| 15 | CH_2_ | 29.7 | 2.23, 2.06 (2H, m) |
| 16 | CH_2_ | 39.57 | 1.61, 1.62 (2H, m) |
| 17 | C | 45.4 | - |
| 18 | CH_3_ | 26.49 | 0.947 (3H, s) |
| 19 | CH_3_ | 11.27 | 0.702 (3H, s) |
| 20 | CH_3_ | 26.76 | 0.952 (3H, s) |
| 21 | CH | 132.14 | 7.493 (1H, s) |

^a^Multiplicities were determined by DEPT experiments.

*^b^*Assignment based on HMQC and HMBC experiments.

**Table S2 Elemental composition of daughter ions of acid degradation product of stanozolol (*m/z* 311).**

| Fragment  (*m/z*) | Proposed formula | Observed mass | Calculated mass | Error (ppm) |
| --- | --- | --- | --- | --- |
| 229 | C_17_H_25_^+^ | 229.1973 | 229.195 | 9.6972 |
| 201 | C_15_H_21_^+^ | 201.1623 | 201.1637 | -7.3436 |
| 189 | C_12_H_17_N_2_^+^ | 189.1369 | 189.1386 | -9.1209 |
| 149 | C_9_H_13_N_2_^+^ | 149.1065 | 149.1073 | -5.5326 |
| 135 | C_8_H_11_N_2_^+^ | 135.0923 | 135.0916 | 4.6273 |
| 107 | C_8_H_11_^+^ | 107.0864 | 107.0855 | 8.1535 |
| 95 | C_5_H_7_N_2_^+^ | 95.0612 | 95.0603 | 8.6815 |
| 81 | C_4_H_5_N_2_^+^ | 81.0448 | 81.0447 | 0.9298 |

**Scheme S1**. Proposed CID-MS/MS fragmentation pathway of acid-degradation product of stanozolol.
